# Unfavourable risk factor control after coronary events in routine clinical practice

**DOI:** 10.1186/s12872-016-0387-z

**Published:** 2017-01-21

**Authors:** Elise Sverre, Kari Peersen, Einar Husebye, Erik Gjertsen, Lars Gullestad, Torbjørn Moum, Jan Erik Otterstad, Toril Dammen, John Munkhaugen

**Affiliations:** 1 Department of Medicine, Drammen Hospital, 3004 Drammen, Norway; 2 Department of Medicine, Vestfold Hospital, Tonsberg, Norway; 3 Department of Cardiology, Oslo University Hospital Rikshospitalet, Medical Faculty, University of Oslo, Oslo, Norway; 4 Department of Behavioural Sciences in Medicine, University of Oslo, Oslo, Norway

**Keywords:** Secondary prevention, Coronary heart disease, Risk factors, Guidelines

## Abstract

**Background:**

Risk factor control after a coronary event in a recent European multi-centre study was inadequate. Patient selection from academic centres and low participation rate, however, may underscore failing risk factor control in routine clinical practice. Improved understanding of the patient factors that influence risk factor control is needed to improve secondary preventive strategies. The objective of the present paper was to determine control of the major risk factors in a coronary population from routine clinical practice, and how risk factor control was influenced by the study factors age, gender, number of coronary events, and time since the index event.

**Methods:**

A cross-sectional study determined risk factor control and its association with study factors in 1127 patients (83% participated) aged 18-80 years with acute myocardial infarction and/or revascularization identified from medical records. Study data were collected from a self-report questionnaire, clinical examination, and blood samples after 2-36 months (median 16) follow-up.

**Results:**

Twenty-one percent were current smokers at follow-up. Of those smoking at the index event 56% continued smoking. Obesity was found in 34%, and 60% were physically inactive. Although 93% were taking blood-pressure lowering agents and statins, 46% were still hypertensive and 57% had LDL cholesterol >1.8 mmol/L at follow-up. Suboptimal control of diabetes was found in 59%. The patients failed on average to control three of the six major risk factors, and patients with >1 coronary events (p < 0.001) showed the poorest overall control. A linear increase in smoking (*p* < 0.01) and obesity (*p* < 0.05) with increasing time since the event was observed.

**Conclusions:**

The majority of coronary patients in a representative Norwegian population did not achieve risk factor control, and the poorest overall control was found in patients with several coronary events. New strategies for secondary prevention are clearly needed to improve risk factor control. Even modest advances will provide major health benefits.

**Trial registration:**

Registered at ClinicalTrials.gov (ID NCT02309255).

**Electronic supplementary material:**

The online version of this article (doi:10.1186/s12872-016-0387-z) contains supplementary material, which is available to authorized users.

## Background

Over the recent years, there has been a decline in mortality rates worldwide [1] leaving a large number of coronary heart disease (CHD) patients in need of optimal secondary prevention. A positive trend in acute myocardial event rates and recurrences from 1994-2009 were also found in Norway [2]. The association between modifiable risk factors and CHD is overwhelmingly documented [3], likewise the benefit of achieving risk factor control to reduce the risk of subsequent events [3, 4]. Despite evidence-based guidelines [5] and cardiac rehabilitation programs for more than 20 years, the EuroAspire studies revealed that the implementation of secondary prevention is far from optimal, with increasing prevalence of smoking in patients <50 years, physical inactivity, obesity and diabetes [6, 7]. In the European cohort of the REACH Study (2003-2004), 40% of symptomatic cardiovascular disease patients had poor control of at least three of the five risk factors assessed [8]. In the Clarify study conducted a decade later, some improvements were found, but even in Europe, the best region, 50% did not achieve risk factor control [9].

Even though the abovementioned studies provide valuable data on the quality of secondary prevention, patient selection could potentially be a matter of concern. In EuroAspire IV [6] patient inclusion was conducted mainly from academic centres, with potentially better secondary prevention than general cardiac practice. Furthermore, the average interview rate was 49%, and the remaining non-participants were probably more likely to have an even poorer risk factor control. In other multinational studies [9–11], patient identification and inclusion has been conducted at outpatient clinics, often specialist centres, and patients attending them may be more concerned about their health. Previous prevalence estimates thus most likely overestimate adherence to guidelines in the general population of CHD patients. Estimates based on studies of everyday clinical practice are clearly needed.

The reasons for unhealthy lifestyle and low risk factor control are complex and poorly understood and the identification of patient and healthcare factors of importance for coronary risk profile remains a public health priority [5]. The overall aim of the The NORwegian CORonary (NOR-COR) Prevention Study is to identify medical, and psychosocial factors associated with unfavourable risk factor control after a cardiovascular event. The present paper determines control of the six major coronary risk factors based in routine clinical practice, and identifies the influence of age, gender, number of coronary events, and time since the index event.

## Methods

### Design and population

The design, methods, and baseline characteristics of the NOR-COR Study have been described elsewhere [12]. Briefly, 1789 consecutive patients aged 18-80 years with a first or recurrent coronary event defined as acute myocardial infarction, coronary artery by-pass graft operation, or percutaneous coronary intervention (PCI) were identified from hospital discharge lists from 2011-14. In patients with recurrent coronary events, the index event was defined as the last event recorded prior to the time of study inclusion. Of these patients, 423 were excluded due to cognitive impairment (*n* = 28), psychosis (*n* = 18), drug abuse (*n* = 10), short life expectancy (*n* = 136), dead (*n* = 160), not able to understand Norwegian (*n* = 44), and other (n = 27). Of the remaining 1366 invited patients, 1127 (83%) participated in attending a clinical visit and completing a comprehensive questionnaire [12] after 2-36 months (median 16) follow-up. The frequency of missing values for the questionnaire based data was low, within the range from 0 - 10%.

The study was conducted at two Norwegian hospitals (Drammen and Vestfold) with a total catchment of 380,000 inhabitants corresponding to 7.4% of the Norwegian population. The catchment area has a representative blend of city and rural districts and is representative of Norwegian geography, economy, age distribution, morbidity, and mortality [13]. The cardiac rehabilitation program at Drammen Hospital includes a multi-disciplinary one day “heart school”, and exercise training twice per week for 6 weeks. The Hospital of Vestfold provides comprehensive lifestyle intervention described elsewhere [14].

### Ethics, consent and permission

The study was approved by the Regional Committee of Ethics in Medical Research. All patients signed a written informed consent prior to study participation.

### Study assessments

Medication and co-morbidity at the index event were registered from the hospital medical records. Cardiovascular medication, risk factors and study factors at follow-up were obtained from the self-report questionnaire, the clinical examination and blood-samples. All blood samples were analysed at Drammen hospital. Diet was assessed by a brief diet questionnaire including seven selected quantitative questions (the frequency of intake of different types of foods and beverages). These questions have been validated against intake of matching food groups [15]. Time since the index coronary event was calculated from index event to the date of study inclusion. Low education was defined by completion of primary- and secondary school only.

### Major coronary risk factors

- *Smoking*: categorized as current, former or never.
- *Overweight* and *obesity:* Body weight was measured in light clothes without shoes (SECA 813, DE). Height was measured using a wall fixed mechanical measuring rod (SECA 264, DE). Overweight and obesity was defined as body mass index (BMI) >25 kg/m^2^ and >30 kg/m^2^, respectively. Waist circumference was measured with a non-stretchable tape (SECA 201, DE). A waist circumference above 94 cm and 102 cm in men and above 80 cm and 88 cm in women was defined as central overweight and obesity, respectively.
- *Physical activity*: assessed by frequency (never, <1 time weekly, 1 time weekly, 2-3 times weekly and almost every day), intensity (light, medium and vigorous), and duration (<15 min, 15-29 min, 30-60 min and >60 min). Low physical activity was defined as less than moderate activity level for 30 min of 2-3 times a week.
- *Blood Pressure (BP) control:* BP was measured after standard procedures using a Welch Allyn digital sphygmomanometer. Unfavourable BP control was defined as BP > 140/90 mmHg (>140/80 mmHg in diabetics).
- *Blood-sugar control:* assessed by HbA1c analysed - Tosoh G8, Ca, US. Unfavourable blood sugar control was defined as HbA1c ≥6.1% (non-diabetics) and >7.0% (diabetics) [5].
- *Low density lipoprotein (LDL) cholesterol:* analysed - Architect ci16200, Ca, US. Elevated LDL cholesterol was defined > 1.8 mmol/l [5].

### Statistics

Statistical analyses have been performed using SPSS version 21. Parametric descriptive statistics were applied. Binary logistic regression analysis was used to calculate odds ratios (ORs) for unfavourable risk factor control and adjusted for age, gender, number of coronary events, and time since the index event.

General Linear Model (ANCOVA) was used to estimate marginal means for number of unfavourable risk factors (smoking, BMI, physical inactivity, BP, LDL cholesterol, and HbA1c) by age, gender and number of coronary events with all independents controlled as dummies simultaneously, and with time since event entered as a linear covariate.

## Results

Baseline characteristics are presented in Table 1. Myocardial infarction and stable CHD was the index event in 80% and 20% of the patients, respectively. Angiography was performed in all patients but one, and 90% were revascularized. Patients with >1 coronary event amounted to 30% with a median number of events of 2 (range 2-11). In this group, the proportion of patients with diabetes was more than twice that seen among those with one event only (28% vs. 12%, p < 0,001).

**Table 1:** Characteristics of the patients (*n* = 1127) at the time of the index coronary event

Mean age at index event (Standard Deviation)	61.6 (9.6)
Women (%)	21
Smoking (%)	35
Diagnoses	
ST-elevation infarction (%)	30
Non ST-elevation infarction (%)	50
Stable or unstable angina (%)	20
More than 1 coronary event (%)	30
Angiographic findings	
No significant stenoses (%)	6
Singel vessel disease (%)	55
Multi-vessels disease (%)	39
Intervention	
PCI^a^ with stent (%)	75
PCI^a^ without stent (%)	2
Coronary artery bypass graft operation (%)	13
No intervention (%)	10
Previous or ongoing participation in cardiac rehabilitation (%)	50
Co-morbidity	
Hypertension (%)	43
Diabetes type I or II (%)	17
Heart failure (%)	13
Atrial fibrillation (%)	9
Stroke or transitory ischemic attack (%)	7
Peripheral artery disease (%)	9
Medication at discharge after the index event	
Aspirin (%)	99
Other antiplateles (%)	88
Statins (%)	96
Beta blockers (%)	85
ACE inhibitors or ARB^b^ (%)	56
Calsium channel blockers (%)	16
Diuretics (%)	22
Antidiabetic (%)	11
Insulin (%)	4
Wafarin or NOAC^c^ (%)	7

All information was obtained from the hospital medical records

^a^Percutaneous coronary intervention, ^b^ACE, angiotensin converting enzyme; ARB, angiotensin receptor blocker. ^c^NOAC, new oral anticoagulants

The prescription rate of recommended preventive drugs [5] was high at discharge. All the patients treated with PCI were prescribed dual anti-platelet treatment. At follow-up, there was a small reduction in the use of beta-blockers (from 85 to 72%) and angiotensin converting enzyme inhibitor (ACEI)/angiotensin receptor blocker (ARB) (from 56 to 50%), while the proportions that used at least one statin (93%) and anti-platelet agent (97%) were almost identical. At the time of follow-up, 50% of the patients had attended cardiac rehabilitation.

**Fig. 1:**
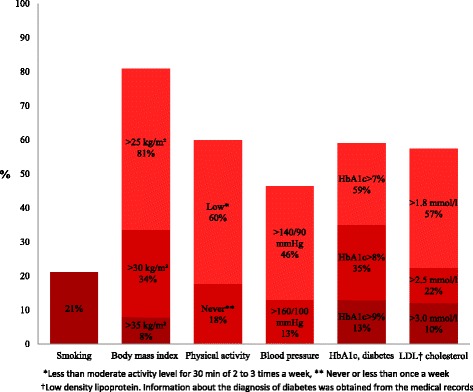
Proportion of coronary risk factors 2-36 months after the index coronary event

The proportion of unfavourable risk factors at follow-up was high (Fig. 1). Of those who smoked at baseline, 56% continued to do so. The majority of patients (84%) had an increased waist circumference, and 60% had central obesity. Ninety-three per cent of the patients used at least one BP lowering drug at discharge after the index event (Table 1), and the same percentage reported use of statin at follow-up. However, the frequency of elevated BP and LDL cholesterol at follow-up were still high. Of the diabetic patients 59% had HbA1c >7% although 79% used blood sugar lowering medication. In patients without known diabetes, 21% had an HbA1c value ≥6.1% and of these patients 8% had HbA1c ≥6.5% indicating diabetes [16]. The proportion that reported to eat fish less than 3 times a week was 46%, while 62% ate fruits or vegetables less than two times daily, and 40% less than once daily.

Current smoking (25% vs. 12%, *p* < 0.001) and physical inactivity (64% vs 34%, *p* < 0.001) were significantly more frequent in patients with low vs. high education, while overweight, unfavorably blood pressure, blood glucose and LDL cholesterol control were not.

**Fig. 2:**
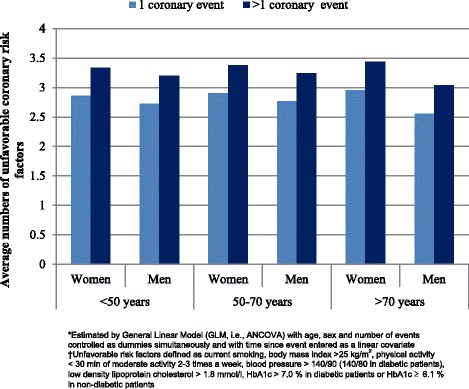
Estimated marginal means* of number of coronary risk factors†

The estimated marginal means for number of unfavourable risk factors [5] by gender, age and number of coronary events are shown in Fig. 2. On average, the patients had three of the six measured risk factors not at target according to guideline recommendations [5]. Less than 2% achieved control for all risk factors, while 62% had three or more unfavourable risk factors. Patients with more than one coronary event (β 0.43, *p* < 0.001) had the poorest overall risk factor control.

Multi-adjusted odds ratios (OR) for unfavourable coronary risk factors at follow-up by age, gender, number of coronary events, and time since the index event are shown in Additional file 1. Current smoking (*p* < 0.001), obesity (*p* < 0.001) and elevated HbA1c (*p* < 0.01) were significantly more frequent in the younger patients, while inadequate BP control (*p* < 0.001) was more frequent with increasing age. ORs for current smoking, low physical activity, and LDL >1.8 mmol/l were significantly higher in women compared to men. ORs for low physical activity, obesity, and elevated LDL cholesterol were significantly higher in patients with several coronary events. There were no significant differences in ORs between the four time groups since the index event, but for smoking (*p* < 0.01) and obesity (*p* < 0.05) the test for linear trend was statistically significant with reduction in risk factor control with increasing time since the event.

## Discussion

Of the CHD patients included from a high income country with a well-developed health care system [17], the majority had a poor risk factor control and thus did not achieve adequate secondary prevention. There were high proportions of current smoking, obesity and physical inactivity. Blood pressure, cholesterol and blood sugar control were inadequate despite the high reported use of medications. Only a minority of patients (<2%) fulfilled the guidelines recommendations [5] for all coronary risk factors, and more than half of them had inadequate control of three or more risk factors. The measured study factors influenced risk factor control with the poorest overall lifestyle control in the youngest patients. Patients with several previous CHD events had the poorest overall coronary risk factor control. There was a higher prevalence of smoking and obesity with increasing time since the since the coronary event.

There are certain limitations of the study. First, the coronary risk factors and study factors were measured at one point in time and thus are prone to measurement and recall bias. Moreover, diet is calculated by a semi-quantitative measure, only. Our questions about physical activity have been validated [18], and we have chosen cut off values as close as possible to guidelines recommendations. Information about the number and the different types of antiplatelet agents at follow-up is not available. The routine clinical setting and the high participation rate (83%) are important strengths of the study. The time span from the index event to follow-up was 2-36 months allowing us to assess how time influences risk factor control. This might impose a selection bias by survival effect. The contribution of excluded patients due to death and short life expectancy is, however, quite similar among the groups with an index event within one year (33%), two years (34%), and three years (33%), respectively, prior to inclusion. Thus, the risk for bias by survival should be minor.

The latest EuroAspire Study [6] had similar inclusion criteria and age distribution as the NOR-COR Study, and in comparison they found a higher proportion of LDL cholesterol >1.8 mmol/l (81% vs. 57%) and diabetes (27% vs. 17%), but fewer diabetic patients had HbA1c >7% (48% vs. 59%). Low physical activity was defined differently, but both studies showed that low physical activity was predominant (60% vs. 60%). The frequencies of hypertension (43% vs. 46%), obesity (38% vs. 34%), and central obesity (58% vs. 60%) were quite similar. The proportion of current smoking was significantly higher (21%) in our CHD population compared to both EuroAspire IV [6] (16%), and other international studies [9–11, 19] (12-18%). Statistics from OECD indicate a lower prevalence in Norway versus average EU regarding smoking (19% vs. 23% [average EU]) [20] and obesity (10% vs. 18% [OECD average]) [21]. It is therefore a paradox that a higher rate of smoking was found among CHD patients in Norway compared to Europe, while the rate of obesity was quite similar. This paradox can be explained by the aforementioned risk of selection bias [6, 7] and by the contribution of non-responders. In the present study with high participation rate from routine clinical practice, these factors are to a higher degree accounted for. There is an ample risk that previous studies [6, 7] have underestimated the prevalence of smoking and obesity in CHD patients.

We found a higher use of anti-platelets (97% vs. 94%), and statins (93% vs. 86%), but lower use of beta-blockers (72% vs. 83%) and ACEI/ARBs (50% vs. 75%) compared with EuroAspire IV [6]. However, there were significant differences in the use of these drugs in various European countries [6].

Large studies from different regions worldwide have also demonstrated that 30-80% of CHD patients had diabetes, were obese, and had LDL cholesterol and BP above the recommended targets [9–11]. In the REACH Registry, one-year risk of subsequent cardiovascular events was inversely related to risk factor control [22], emphasizing the importance of reaching these treatment goals.

The reasons for the low adherence to secondary prevention are complex and multi-factorial [5, 23]. Low socioeconomic status is known to affect both risk factor control and the course of CHD negatively [24, 25], and we confirmed the well-known association between low education and unfavourable lifestyle. Psychosocial factors such as anxiety, depression, type-d personality and lack of social support may affect both etiological factors, lifestyle and adherence, and are associated with adverse outcomes in CHD patients [26]. Furthermore many revascularized patients have no symptoms. In a recent post PCI study, many patients perceived that they were cured from their CHD [27]. Few reported lifestyle-style factors as being causal, and almost 40% perceived no need for lifestyle changes. Patients’ understanding of CHD and CHD risk factors have been shown to be insufficient [28]. Furthermore, many patients attribute their disease to factors they cannot influence [27] like age and family history, that may partly explain lack of motivation to change lifestyle and adhere to their medical regimen. Despite overwhelming documentation of the benefits of secondary preventive drug [5], a meta-analysis revealed that only 60% of CHD patients had good adherence to cardiovascular medication [29]. Poor adherence with medication may in part explain why many patients do not reach treatment targets for BP, cholesterol and blood sugar. When the vast majority of patients were prescribed cholesterol and BP lowering drugs, and only 40-55% reached treatment targets, it is possible that the drugs chosen were not the optimal, the dosages applied were too low, the patients were not compliant or a combination. The clinical significance of long-term dual anti-platelet therapy after coronary stent procedures was recently documented in CHD [30], reflecting the need for improved secondary prevention programs that also address drug-adherence reliability and over time (>12 months).

The youngest patients had the highest proportion of unfavourable lifestyle factors, and this might have contributed to an early onset of CHD. The positive trend in acute myocardial infarction event rates and recurrences from 1994-2009 in Norway were mostly seen among patients older than 65 years, whereas less favourable trends were observed among younger patients, in particular women [2]. This is concerning, and may be due to the particular poor risk factor control in this sub-group as demonstrated in the present study. Correspondingly, the poor risk factor control in patients with more than one coronary event might be why they suffer repeated events. It is concerning that the success of secondary prevention in these high-risk patients is that poor. The effect of lifestyle intervention programs on risk factor control and subsequent events is well documented [14, 31]. In the present study, only half of the patients attended the available programs. The participation rates in cardiac rehabilitation programs range between 30-60% in Europe, lowest among the oldest patients, and those with co-morbidity [7, 32]. Underutilization of effective preventive programs or implementation of programs that do not result in adherence in routine clinical practice, may contribute to poor risk factor control. The higher proportion of current smokers and obese patients with increasing time since the coronary event underline the need for more long-lasting secondary preventive programs [33].

Medical and psychosocial factors may act as barriers to lifestyle changes, treatment adherence and may moderate the effects of cardiac rehabilitation [26]. The predictors of good adherence to risk factor control are likely to differ by patient characteristics and risk factors, indicating a need for more tailored interventions [34]. Accordingly, we found different impact of age, gender, education, time since the event, and the number of events on the major risk factors. In the further studies, we aim to explore the relative importance of a number of potentially modifiable factors on risk factor control [12].

## Conclusion

The majority of CHD patients from a routine clinical practice in a representative Norwegian population did not achieve control of the major coronary risk factors. The measured non-modifiable study factors had different impact on the risk factors, and the poorest overall control was found in patients with several coronary events. It is concerning that secondary prevention of CHD fails in a country with a well-developed health care system. Further knowledge about factors associated with poor risk factor control and strategies for implementation of these factors are strongly needed to improve secondary prevention. Even modest advances will provide major health benefits.

## Additional file

Additional file 1: Multi-adjusted odds ratio for unfavourable coronary risk factors 2-36 months after the index coronary event. (DOCX 16 kb)

## Abbreviations

ACE: Angiotensin converting enzyme
ARB: Angiotensin receptor blocker
BMI: Body mass index
BP: Blood pressure
CHD: Coronary heart disease
CI: Confidence interval
LDL: Low density lipoprotein
NOAC: New oral anticoagulant
OR: Odds ratio
PCI: Percutaneous coronary intervention
