# Supplementary material for: Unfavourable risk factor control after coronary events in routine clinical practice
Source: BMC Cardiovasc Disord. 2017 Jan 21;17:40. doi: 10.1186/s12872-016-0387-z (PMC5251244; doi:10.1186/s12872-016-0387-z)
Supplement: Additional file 1: — Multi-adjusted odds ratio for unfavourable coronary risk factors 2-36 months after the index coronary event. (DOCX 16 kb) [file 12872_2016_387_MOESM1_ESM.docx]

**Additional file 1:** Multi-adjusted odds ratio for unfavourable coronary risk factors 2-36 months after the index coronary event

|  | Current smoking (n=230) | | Body Mass Index >30 kg/m^2^ (n=340) | | Low physical activity** (n=665) | | Blood Pressure† >140/90 mmHg (n=470) | | HbA1c†† >7.0% (n=108) | | LDL cholesterol††† >1,8 mmol/l (n=628) | |
| --- | --- | --- | --- | --- | --- | --- | --- | --- | --- | --- | --- | --- |
|  | % | OR (CI)* | % | OR (CI)* | % | OR (CI)* | % | OR (CI)* | % | OR (CI)* | % | OR (CI)* |
| Age |  |  |  |  |  |  |  |  |  |  |  |  |
| < 50 years (n=151) | 26 | 1.00 | 47 | 1.00 | 55 | 1.00 | 31 | 1.00 | 76 | 1.00 | 61 | 1.00 |
| 50-69 years (n=730) | 23 | 0.82 (0.54-1.24) | 34 | 0.56 (0.38-0.82) | 58 | 1.06 (0.74-1.51) | 48 | 1.99 (1.33-2.99) | 59 | 0.36 (0.13-1.01) | 58 | 0.82 (0.56-1.18) |
| ≥ 70 years (n=246) | 14 | 0.44 (0.26-0.75) | 23 | 0.32 (0.20-0.52) | 70 | 1.72 (1.11-2.65) | 53 | 2.43 (1.53-3.89) | 51 | 0.29 (0.09-0.93) | 54 | 0.66 (0.43-1.02) |
| Gender |  |  |  |  |  |  |  |  |  |  |  |  |
| Women (n=237) | 25 | 1.00 | 36 | 1.00 | 69 | 1.00 | 41 | 1.00 | 51 | 1.00 | 66 | 1.00 |
| Men (n=890) | 20 | 0.68 (0.48-0.97) | 33 | 0.80 (0.58-1.11) | 58 | 0.60 (0.44-0.82) | 48 | 1.29 (0.95-1.77) | 62 | 1.91 (0.91-4.01) | 55 | 0.57 (0.42-0.78) |
| Time since the index event |  |  |  |  |  |  |  |  |  |  |  |  |
| 2-6 months (n=233) | 19 | 1.00⁺ | 29 | 1.00⁺ | 61 | 1,00 | 48 | 1.00 | 59 | 1.00 | 53 | 1.00 |
| 6-12 months (n=242) | 18 | 0.91 (0.56-1.46) | 32 | 1.20 (0.79-1.81) | 58 | 0.81 (0.56-1.19) | 48 | 0.98 (0.67-1.44) | 61 | 1.05 (0.40-2.78) | 58 | 1.22 (0.84-1.77) |
| 12-24 months (n=347) | 21 | 1.12 (0.73-1.72) | 34 | 1.37 (0.93-2.00) | 60 | 0.96 (0.67-1.36) | 48 | 0.99 (0.69-1.41) | 65 | 1.35 (0.55-3.29) | 61 | 1.44 (1.02-2.03) |
| 24-36 months (n=305) | 26 | 1.48 (0.97-2.28) | 37 | 1.46 (0.99-2.16) | 60 | 1.03 (0.72-1.48) | 42 | 0.80 (0.55-1.16) | 52 | 0.69 (0.27-1.75) | 57 | 1.23 (0.86-1.75) |
| Numbers of coronary events |  |  |  |  |  |  |  |  |  |  |  |  |
| 1 event (n=790) | 20 | 1.00 | 32 | 1.00 | 56 | 1.00 | 44 | 1.00 | 62 | 1.00 | 55 | 1.00 |
| > 1 event (n=337) | 23 | 1.36 (0.99-1.87) | 37 | 1.34 (1.01-1.80) | 69 | 1.77 (1.34-2.34) | 52 | 1.31 (1.00-1.73) | 57 | 0.75 (0.40-1.42) | 64 | 1.58 (1.20-2.08) |

*CI=95% confidence interval, ** Less than 30 minutes of moderate activity 2-3 times a week,

† blood pressure > 140/90 (140/80 in diabetic patients), †† in diabetic patients, †††LDL, low density lipoprotein cholesterol

⁺ Significant linear test for trend in current smoking and BMI <30 kg/m^2^, respectively, using time since the coronary event as continuous variable.

All estimates are adjusted for age, gender, time since the index event and number of coronary events.
